# The Effects of *Naematelia aurantialba* on the Pasting and Rheological Properties of Starch and the Research and Development of Soft Candy

**DOI:** 10.3390/foods13020247

**Published:** 2024-01-12

**Authors:** Yanfen Cheng, Cuixin Su, Shijie Wei, Jing Zhao, Fen Wei, Xiaolong Liu, Hanbing Wang, Xiaoyue Wu, Cuiping Feng, Junlong Meng, Jinling Cao, Shaojun Yun, Lijing Xu, Xueran Geng, Mingchang Chang

**Affiliations:** 1 College of Food Science and Engineering, Shanxi Agricultural University, Jinzhong 030801, China; 17735461182@163.com (C.S.); sjbx1998@163.com (S.W.); 18536497670@163.com (J.Z.); wf2945567053@163.com (F.W.); liuxiaolongsxu@163.com (X.L.); wanghb0422@163.com (H.W.); 13111183585@163.com (X.W.); ndfcp@163.com (C.F.); mengjunlongseth@hotmail.com (J.M.); caojinling7928@163.com (J.C.); yunshaojun@163.com (S.Y.); xulijing383942909@163.com (L.X.); gengxueran2007@163.com (X.G.); 2 Shanxi Edible Fungi Engineering Technology Research Center, Jinzhong 030801, China; 3 Shanxi Key Laboratory of Edible Fungi for Loess Plateau, Jinzhong 030801, China

**Keywords:** *Naematelia aurantialba*, starch, processing characteristics, soft candy, physicochemical properties

## Abstract

To study the effects of Naematelia aurantialba (NA) on the rheological and gelatinization properties of starch, the processing methods of NA were diversified. In this study, the gelatinization and rheological properties of corn starch (CS) and edible cassava starch (ECS) were investigated by adding NA with different mass fractions. Starch soft candy was prepared using NA, CS, and ECS as the main raw materials. Rheological studies showed that both CS-NA and ECS-NA exhibited elastic modulus (G′) > viscosity modulus (G″), implying elastic behavior. G′ was such that CS+1%NA > CS+5%NA > CS+3%NA > CS > CS+2%NA > CS+4%NA > ECS+4%NA > ECS+3%NA > ECS+5%NA > ECS+2%NA > ECS+1%NA > ECS. The gelatinization implied showed that after adding NA, the pasting temperature of CS-NA and ECS-NA increased by 1.33 °C and decreased by 2.46 °C, while their breakdown values decreased by 442.35 cP and 866.98 cP, respectively. Through a single-factor test and orthogonal test, the best formula of starch soft candy was as follows: 0.4 f of NA, 10 g of white granulated sugar, a mass ratio of ECS to CS of 20:1 (g:g), 0.12 g of citric acid, 1 g of red date power, and 16 mL of water. The soft candy was stable when stored for two days. This study offers a new direction for the research and development of NA starch foods.

## 1. Introduction

*Naematelia aurantialba* (NA) is also known as yellow fungus or gold tremella because of its golden yellow color. It belongs to Fungi, Basidiomycetes, Tremella, Argyria, and Auriculariaceae [1]. It is a gelatinous fungus with a delicate taste [2] and is rich in polysaccharides, protein, vitamins, beta-carotene, iron, zinc, etc. [3]. The polysaccharide content of NA is as high as 75.30% [2]. It has various biological activities such as antiaging, relieving cough, eliminating phlegm, and enhancing immunity [4]. Sun et al. [5] developed NA yogurt due to its rich protein and antiaging biological activities. After adding NA, the product had a good curd effect and demonstrated the unique fleshiness and fragrance of NA. Sun et al. [4] developed a new type of solid drink with beneficial qi-nourishing lung functions due to the effect of NA on eliminating phlegm and relieving cough. Hao et al. [6] researched and developed a compound jam because NA had functions, such as clearing lung heat and relieving cough, and could be used for skincare, and anti-wrinkling. The compound jam added with NA had a natural NA flavor and can maintain long-term stability. Sun et al. [4] created a new soybean beverage because of the flavor and color of NA. After adding NA, the bitterness was reduced, and the sour taste increased while maintaining the sweetness of the unfermented soybean beverage. At present, the development of NA-related foods has mainly focused on beverages, such as yogurt and soybean drinks, and there are few studies on “NA-starch” foods. He [7] et al. added NA when making bread and found that NA can reduce its hardness and improve elasticity, making bread soft and sturdy, but its mechanism has not been studied. The NA glycopeptide capsules developed by Meng et al. [8] due to the efficacy of NA in reducing phlegm and relieving cough have the efficacy of improving body immunity and preventing and treating hepatitis. To advance the research and development of NA-related healthcare products, the development of a soft candy suitable for public beauty has become the focus of much research.

Corn starch (CS) and edible cassava starch (ECS) are commonly used starches and are available at low cost. They are widely applied in soft candies, pastries, puddings, taro balls, and other products. However, due to the inherent defects of poor stability, easy aging, and easy expansion, natural starch cannot meet the processing requirements of some foods [9]. At present, the complex system of starch and non-starch polysaccharides has received extensive attention. Many studies have shown that the combination of non-starch polysaccharides with different starches can significantly affect the gelatinization, rheological, and digestion characteristics of the complex system. Studies found that polysaccharides [10], xanthan gum, guar gum, locust bean gum [11], and sodium alginate [11] can all promote the gelatinization of CS and enhance the viscoelasticity of the complex system. However, there was, at the time of writing, no research report on the combination system of NA with CS and ECS.

It was assumed that NA can affect the rheological and gelatinization characteristics of starch so that the processing methods of NA can be diversified. Therefore, the rheological and gelatinization properties of CS-NA and ECS-NA complexes were obtained in this study. Moreover, further study of the processing properties of CS-NA and ECS-NA complexes provides a theoretical basis for the development of soft candy. A single-factor test and orthogonal test were used to optimize the formula of soft candy. The surface of the soft candy was smooth, the color was golden, and the taste was soft and elastic. Additionally, the effects of different storage times on the texture characteristics of the soft candy were studied. This study aimed to expand the range of application range of CS and ECS in food and provide a theoretical basis for the innovation of “NA-starch type” products.

## 2. Materials and Methods

### 2.1. Materials and Reagents

NA powder (available on the market, NA), CS (available on the market, CS, 85.2%), ECS (Honghao Starch Development Co., Ltd., Nanning, Guangxi, China, ECS, 82.8%), red date powder (Duomei Food Co., Ltd., Weifang, Shandong, China), white granulated sugar (Gengma Nanhua Sugar Industry Co., Ltd. Lincang, Yunnan, China), and citric acid (Want Want Food Additives Co., Ltd., Jinan, Shandong, China) were of food grade.

A rapid viscosity analyzer (RVA, BosinTech, RAPID-20), a differential scanning calorimeter (DSC, Mettler Toledo, DSC 3), a texture analyzer (FTC Corporation, TPA, TMS-Pro), and a rheometer (MCR102) were used.

### 2.2. Preparation of NA Powder (NA)

The dried NA specimens were milled (Multifunctional mill, Hongjingtian Industry and Trade Co., Ltd., Jinhua, Zhejiang, China. DE-500g) and passed through a 100-mesh screen to obtain NA.

### 2.3. Rapid Viscosity Analysis (RVA)

CS and ECS have similar actions as a thickener and can improve the gelatinization performance of starch. Therefore, the gelatinization behaviors were investigated using a conventional RVA according to the method of Nawab et al. [12], with minor modifications. Samples (3 g) were weighed in a canister, followed by the addition of 25 mL of distilled water, and 1%, 2%, 3%, 4%, and 5% NA (based on starch) were mixed with two starches (CS and ECS *w*/*w*), respectively. The starch suspension without NA was used as the control group. After being fully pre-stirred with a small plastic propeller, the aluminum crucible was stuck into an RVA in the rotating tower for testing. The specific parameters were as follows: at 50 °C for 1 min, heating at 12 °C/min to 95 °C, constant temperature at 95 °C for 2.5 min, and cooling at 12 °C/min to 50 °C. The stirring procedure is described as automatic stirring at 960 rpm for 10 s and then stirring at 160 rpm until the end of the experiment. The peak viscosity, pasting temperature, setback, breakdown, trough minimum viscosity, and final viscosity were calculated using RVA to study and analyze the gelatinization properties of the complex.

### 2.4. DSC

CS and wheat starch are both cereal starches. So, they were slightly modified according to the research method of He et al. [13] CS and ECS (5 mg each) were weighed, and the added amount of NA was 20%, 40%, and 60% (*w*/*w*) of the mass of starch, respectively, and the resultant was mixed evenly. The sample was added to an aluminum crucible and analyzed using DSC. The heated procedure was from 25 °C to 120 °C at a rate of 10 °C/min, and the empty crucible was used as a reference. DSC curves of CS-NA and ECS-NA complex systems were plotted to study their thermal properties.

### 2.5. Rheological Measurements

Upon the completion of an RVA test 2.3, the fully gelatinized samples were immediately placed in the rheometer on the measurement platform. A cone plate mold with a diameter of PP50 was selected with a 1 mm gap, and the samples were then transferred to a plate and balanced at 25 °C for 30 s before measurement.

#### 2.5.1. Steady Shear Test

Steady-state shear scanning tests were conducted at shear rates ranging from 0.1 s^−1^ to 100 s^−1^ to determine the relationship between the apparent viscosity and shear rate. The effect of the shear rate on the apparent viscosity of CS-NA and ECS-NA was determined.

#### 2.5.2. Dynamic Frequency Test

The elastic modulus (G′), viscosity modulus (G′′), and loss factor (tan δ = G″/G′) were obtained by oscillating frequency scanning with a strain of 1% CS and 10% ECS (in the linear viscoelastic region) in the angular frequency range from 0.1 rad/s to 100 rad/s [14]. The effects of angular frequency on the energy storage modulus (G′), loss modulus (G″), and loss coefficient (tan δ) of CS-NA and ECS-NA were measured.

### 2.6. Preparation of Soft Candy

A total of 14 g of white granulated sugar and 0.12 g of citric acid were taken, and 16 mL of water was added before boiling. The specimens were heated and stirred to dissolve the sugar and citric acid. When the particles were completely dissolved in water, the heating was stopped. The dissolved sugar and acid mixture could be cooled to 80–90 °C. Then, 0.4 g of NA, 1 g of red date powder, 8 g of ECS, and 0.4 g of CS were added, mixed well, and placed in boiling water for 2–3 min to obtain the slurry solution. The sample was heated into the mold and heated over medium heat for 4 min. The cooked NA jujube compound soft candy was cooled at room temperature for about 30 min, and the finished product was obtained after demolding.

#### 2.6.1. Single-Factor Test

On the basis of the pretest, different amounts (0 g, 0.4 g, 0.8 g, 1.2 g, 1.6 g, 2.0 g) of NA could be added, respectively. White granulated sugar (8 g, 10 g, 12 g, 14 g, 16 g), the ratio of ECS to CS (14:1, 16:1, 20:1, 23:1, 26:1 *w*/*w*), and citric acid content (0.08 g, 0.10 g, 0.12 g, 0.14 g, 0.16 g) were set as single variables. A single-factor test was performed to analyze the sensory score and texture determination index under each variable condition.

#### 2.6.2. Orthogonal Experimental Design

On the basis of the single-factor experiment, three optimal levels were selected for each of the four factors: the supplemental level of NA (A), the supplemental level of white granulated sugar (B), the ratio of ECS to CS (C), and the dosage of citric acid (D), and other variables were controlled. Taking sensory evaluation as the investigation index, the orthogonal experimental results were reasonably analyzed and demonstrated, and the best formula for soft candy was selected. L9 (34) orthogonal experiments were performed, regardless of the interaction between the factors. Orthogonal test factors and horizontal designs are shown in Table 1.

### 2.7. Sensory Evaluation of Soft Candy

Three healthy men and three healthy women were invited to evaluate the color, sourness and sweetness, flavor, texture, and taste of the compound soft candy. The sensory scoring standards of NA and red date compound gummies are listed in Table 2.

### 2.8. Textural Measurements

The quality of soft candy was determined by texture analysis. The test conditions were as follows: a 6 mm cylindrical probe was used, the force induction element range was 100 N, the probe was raised to a height of 33 mm on the sample surface, the extrusion distance was 5 mm, the starting detection speed was 60 mm/min, the starting force was 0.3 N, the interval between two extrusions was 3 s, and the measured speed was 200 mm/min. The hardness, springiness, gumminess, and chewiness parameters were determined to study the textural characteristics of soft candy.

### 2.9. Effect of Storage Time on the Texture of Soft Candy

The soft candy samples were prepared according to the best formula selected by single-factor and orthogonal tests, and the samples were sealed and placed at room temperature. The texture of the samples was determined every 24 h and tested for five days to explore the effect of storage time on the quality of soft candy. The methods for the determination of the texture properties of soft candy at different storage times were consistent with those described in Section 2.8.

### 2.10. Statistical Analysis

The experimental data were analyzed by one-way ANOVA using SPSS statistical software (Version 27), and a difference was deemed statistically significant at *p* < 0.05. The results were expressed as the mean ± standard deviation, with significant differences in different superscript letters, and each experiment was repeated three times. Plotting was performed using MS-Excel^®^ 2019 and Origin software (Version 2022).

## 3. Results and Discussion

### 3.1. Effect of the Addition of NA on the Gelatinization Characteristics of CS and ECS

As shown in Table 3, NA significantly increased the peak viscosity, trough minimum viscosity, and final viscosity of CS-NA, and the increase in viscosity was positively correlated with the amount of NA, which was attributed to the effect of polysaccharides in NA. Previous studies have also shown that polysaccharides can increase the viscosity of starch pastes [15]. The interaction between the dissolved starch chains also improved the viscosity of the complex system to some extent. Compared with CS, after the addition of NA, the breakdown value decreased. Starch pastes became more stable and better resistant to heat treatment and mechanical shearing during food processing [16]. The setback value was significantly decreased (*p* < 0.05), and the short-term setback value of CS was inhibited. This was due to the competition between NA and starch in the adsorption system and the hydrogen bonding of NA and permeable amylose, which inhibited the mobile rearrangement of starch molecules and reduced the setback value [17]. From the perspective of application, the effect of NA on the long-term setback of starch warranted further investigation to develop starch-based products with good taste and a longer shelf life [18]. The pasting temperature reflected the difficulty in determining the degree of starch gelatinization. Compared with CS, the pasting temperature with 1%, 2%, 3%, and 4% NA was reduced, while the gelatinization temperature with 5% NA was significantly increased (*p* < 0.05). This was because a small number of polysaccharides in NA had little spatial obstruction to water molecules, and polysaccharides filled the complex system, generated a good bridge between starch particles, improved the affinity between starch particles and water molecules, and promoted the formation of a starch gel network [19,20].

As shown in Table 3, compared with ECS, NA significantly decreased the peak viscosity, trough minimum viscosity, and final viscosity of ECS-NA, and the reduction in viscosity was positively correlated with the amount of NA added. On the one hand, the reason was that due to the large content of ECS amylose, with the increase in the ECS ratio, amylose and amylopectin were entangled in the starch crystallization area, and amylose restricted the expansion of amylopectin and thus inhibited starch pasting, resulting in a decrease in the peak viscosity [21]. On the other hand, the results showed that the polysaccharide in NA inhibited the ability of the starch to expand. This was because NA has strong water absorption, and NA and starch particles in the pasting process compete for water molecules at the same time, resulting in a small expansion of the starch particles [22], which results in a decrease in peak viscosity. This result was similar to Yin et al [23]. Compared with ECS, NA also significantly reduced the setback and breakdown values. The smaller the breakdown value, the stronger the thermal stability of the starch paste. The reason was that the compound system can maintain the integrity of starch particles under the action of heating and shear force, so the backdown value decreased [24]. The setback value reflected the aging degree of starch paste and the strength of the ability to form a gel. The greater the setback value, the stronger the gel, and the more likely it was to age. Therefore, the addition of the 5% NA complex had a stronger ability to form a gel and was easier to age. The pasting temperature decreased with increasing NA, indicating that the ECS gelatinized the mixed system to a greater extent. This result was the same as the trend of the gelatinization of blends of wheat starch, potato starch, and pea starch found by Li et al. Because the gelatinization temperature of CS+5%NA and ECS+5%NA was low and the breakdown value was small, it was easier to gelatinize, and its stability was good.

### 3.2. Analysis of the Thermal Characteristics of CS and ECS with the NA Addition

**Figure 1:**
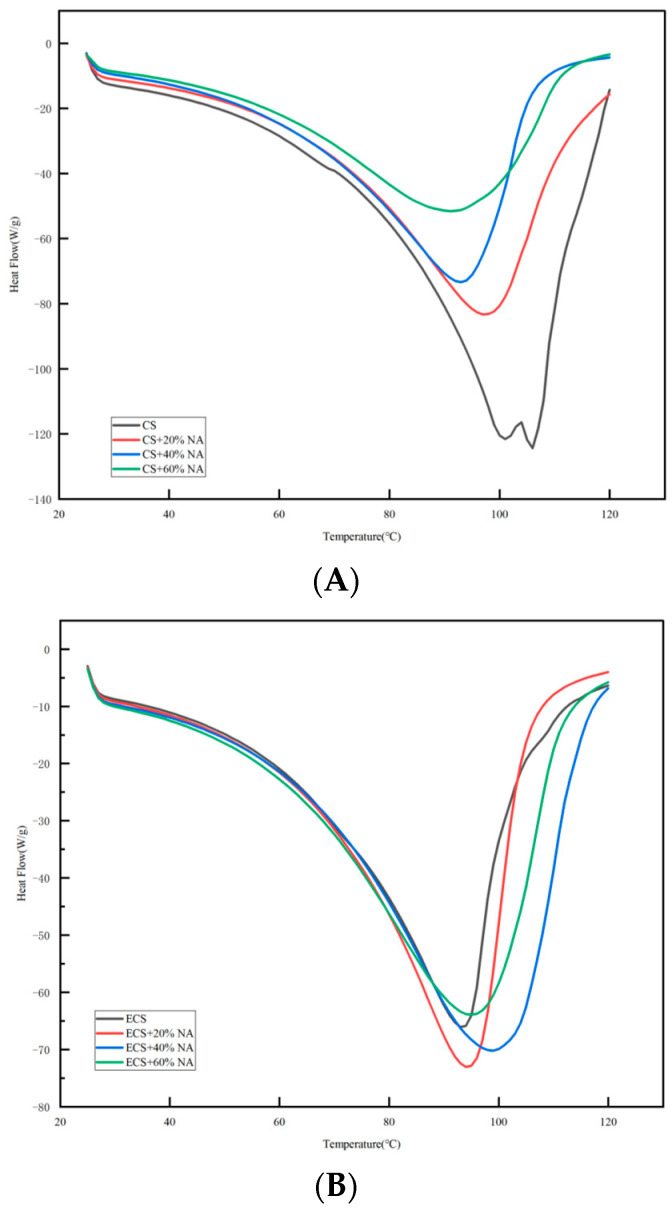
(**A**,**B**) DSC curves of CS-NA and ECS-NA complex systems. Notes: CS: corn starch; ECS: edible cassava starch; NA: *Naematelia aurantialba* powder.

As shown in Figure 1A,B, all CS-NA and ECS-NA complex systems exhibited a single heat-absorption peak. The starting gelation temperature (To) of CS-NA decreased from 100 °C to 90 °C, and the peak gelatinization temperature (TP) decreased with the increasing addition of NA to 101 °C, 98 °C, 93 °C, and 91 °C, respectively. This was because the reaction between NA and amylopectin accelerated the degradation of the crystalline structure of the particles, reduced the resistance of water molecules to enter the starch particles, and thus decreased the values of To and Tp. This matched the conclusion reached by Kong et al. [25] when studying the effect of single glycerol on the thermal properties of waxy maize. However, the starting gelation temperature (To) and peak gelation temperature (TP) of the ECS-NA complex system increased with the increasing addition of NA. The TP was a maximum of 99 °C. This was similar to the experimental results obtained by Luo et al. [19]. This was ascribed to the addition of non-starch polysaccharides resulting in reduced free water of starch for gelation transition and weakened hydration of the amorphous region of starch granules, which resulted in increased starch gelation temperature with a stronger effect with partially reduced availability of water [26]. The gelatinization temperature (TP) was also confirmed by RVA.

### 3.3. Analysis of Rheological Properties of CS and ECS with the Addition of NA

#### 3.3.1. Analysis of Static Rheological Characteristics of CS and ECS with the Addition of NA

**Figure 2:**
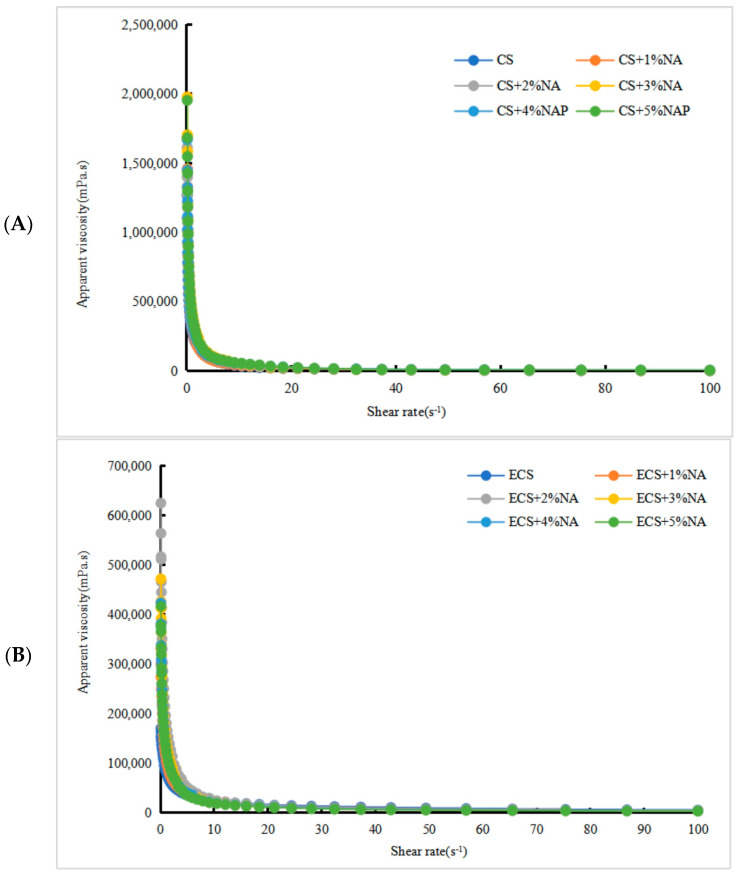
(**A**,**B**) The effect of the NA addition amount on the apparent viscosity of CS and ECS. Notes: CS: corn starch; ECS: edible cassava starch; NA: *Naematelia aurantialba* powder.

As shown in Figure 2A,B, the apparent viscosity of the CS-NA and ECS-NA complex systems both showed a decreasing trend with increasing shear rate, evincing shear thinning (a typical non-Newtonian fluid behavior). The apparent viscosity did not change significantly with the increased NA dose. In the range of 0.1 to 1 s^−1^, the viscosity showed a significant decreasing trend; the viscosity decreased slowly at a rate ranging from 1 to 100 s^−1^. When the shear rate increased, the viscosity decreased gradually. This was because the structure of starch particles was chain-like and relatively scattered. When the scattered chain-like particles were subjected to shear force, the interaction between the particles was reduced, and the phenomenon of shear thinning was observed. This was consistent with the research results of Sun et al. [27]. The viscosity curves of CS-NA and ECS-NA showed an upward trend with the increase in NA addition. In particular, the changes in the viscosity of CS+5%NA and ECS+5%NA were the most significant.

#### 3.3.2. NA Analysis of the Dynamic Rheological Characteristics of CS and ECS

**Figure 3:**
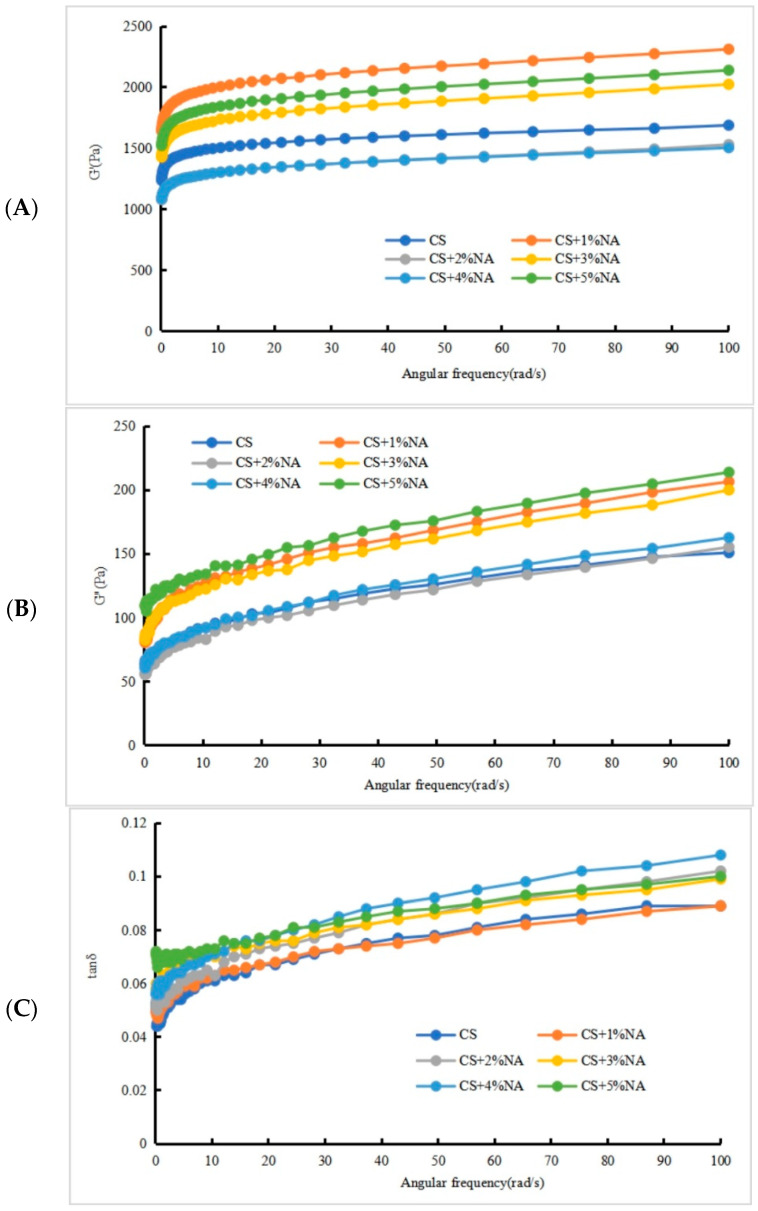

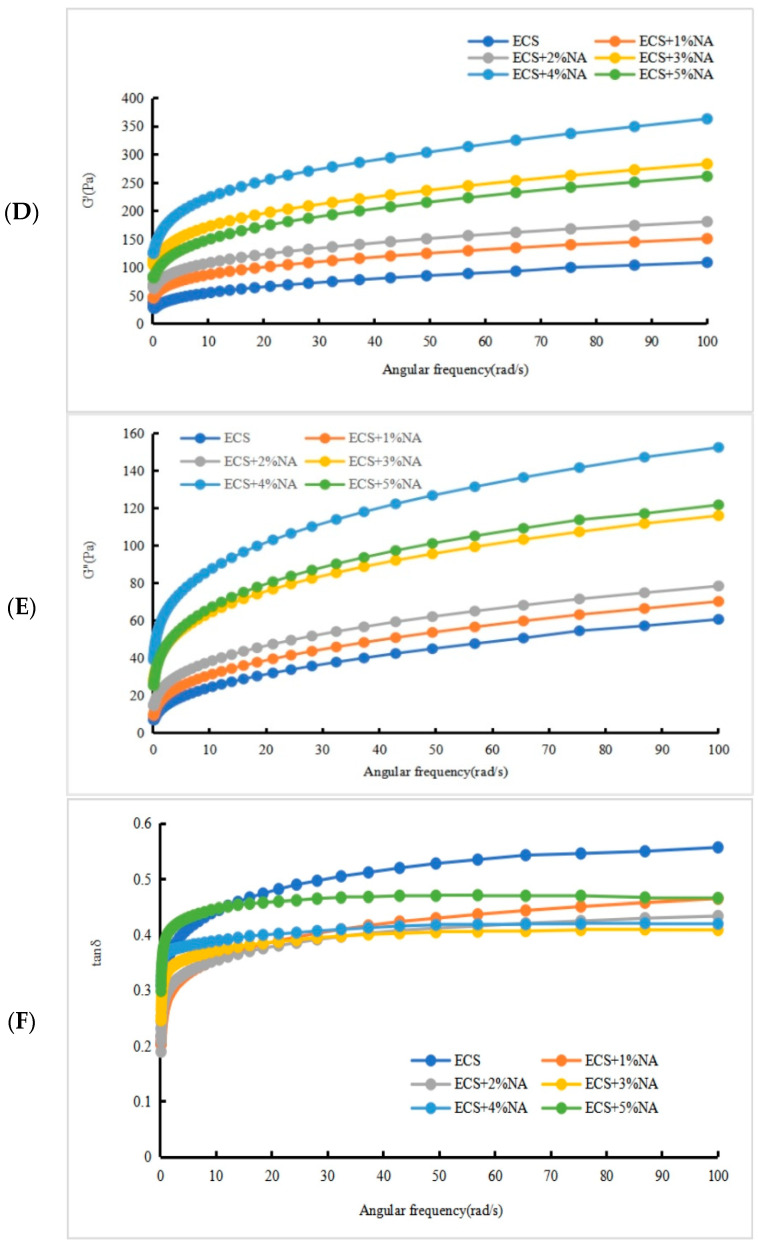
(**A**–**C**) The relationship between the NA addition amount and CS, frequency and modulus and loss coefficient; (**D**–**F**) the relationship between the NA addition and ECS, frequency and modulus and loss coefficient. Notes: CS: corn starch; ECS: edible cassava starch; NA: *Naematelia aurantialba* powder.

As shown in Figure 3A,B,D,E, the CS-NA and ECS-NA complex systems were in the range of 0.1–100 rad/s, and their elastic modulus (G′) and viscosity modulus (G″) increased with increasing angular frequency, showing a frequency dependence and a typical weak gel dynamic rheological spectrum [28]. In CS-NA and ECS-NA complex systems, G′ > G″, indicating that the gels formed by the tested samples were weak gels, and all the systems were largely elastic. After the addition of NA, the values of G′ and G″ of all the compound systems were basically higher than the original starch. The increase in G′ indicated that the addition of NA increased the entanglements between the molecular chains in the starch and NA systems and strengthened the network structure of the gel system, indicating that the CS-NA and ECS-NA systems form a three-dimensional network structure stronger than the pure starch system [29]. The increase in G″ indicated that the thickening properties of polysaccharides in NA improved the viscoelasticity of continuous phases in the CS-NA and ECS-NA systems, indicating that NA plays a decisive role in the rheological properties of CS-NA and ECS-NA. The results of Zhang et al. [30] are also similar.

The tangent of the loss angle (tan δ) was the ratio of G″ to G′. The larger the tan δ was, the greater the viscosity ratio and the stronger the fluidity of the system; otherwise, the greater the elasticity ratio [31]. As shown in Figure 3C,F, tan δ in both CS-NA and ECS-NA complex systems increased with angular frequency; tan δ was less than 1, evincing that the higher the elasticity of the specimen under test. The solid properties of both the CS-NA and ECS-NA complex systems were stronger. The tan δ value of the ECS-NA system decreased with increasing NA addition, and the ECS-NA system showed stronger elastic properties with the addition of NA. The polysaccharide content in NA in water increased, the force of the molecules between polysaccharide and starch increased, the polysaccharide starch gel network structure was formed, and the elasticity and rigidity of the ECS-NA system were enhanced. The results are similar to Cao et al. [32]. In brief, when NA > 5%, the viscoelasticity of CS-NA and ECS-NA was better.

### 3.4. Analysis of the Single-Factor Experiment

#### 3.4.1. Effect of the NA Addition on the Quality of Soft Candy

As shown in Table 4, with an increase in the amount of NA added, the sensory score and springiness of soft candy first increased and then decreased. When the amount of NA added was 0.4 g, the sensory score was the highest, the hardness, springiness, gumminess, and chewiness of the soft candy were better, and the taste and flavor were the best. Therefore, the optimal addition amount of NA was determined to be 0.4 g.

#### 3.4.2. Effect of the Addition of White Granulated Sugar on the Quality of Soft Candy

As shown in Table 4, with an increasing content of white granulated sugar, the sensory score, springiness, and chewiness of soft candy first increased and then decreased. When the amount of white granulated sugar reached 12 g, the sensory score was the highest, the hardness and chewiness were moderate, and the springiness and gumminess were good. Therefore, the optimal amount of white granulated sugar added was determined to be 12 g.

#### 3.4.3. Effect of the Ratio of ECS and CS on the Quality of Soft Candy

As shown in Table 4, with an increase in the ECS supplemental level, the sensory score and chewiness of soft candy first increased and then decreased. Therefore, the optimal addition amount of ECS:CS was 20:1 (*w*/*w*).

#### 3.4.4. Effect of the Citric Acid Addition on the Quality of Soft Candy

Citric acid is a commonly used sour agent in food processing. As shown in Table 4, adding it to soft candies cannot only improve the taste of soft candy but also enhance appetite. With an increase in citric acid added, the sensory score and springiness showed a trend of first increasing and then decreasing. When the amount of citric acid was 0.12 g, the sensory score was the highest, and the springiness and taste were better; therefore, the optimal dosage of citric acid was 0.12 g.

### 3.5. Orthogonal Test Results

The orthogonal experiment results are listed in Table 5. The hardness, resilience, and chewiness had the same degree of influence, and the order of influence was as follows: A > B > D > C (i.e., the addition of NA > the white granulated sugar addition > the citric acid addition > the ratio of ECS and CS). The main and secondary factors affecting springiness were B > A > D > C. The factors affecting the sensory score of soft candy were as follows: A > B > C > D. The springiest formula combination was A_3_B_3_C_1_D_3_. The largest chewiness formula combination was A_3_B_3_C_1_D_1_. The hardest formula combination was A_3_B_2_C_3_D_1_. The gummiest formula combination was A_3_B_3_C_1_D_1_. According to sensory evaluation and texture characteristics, A_2_B_2_C_1_D_2_ was the optimal combination of soft candy, which involved the amount of NA being 0.4 g, the amount of granulated sugar being 12 g, the ratio of ECS and CS being 17:1, and the amount of citric acid being 0.12 g.

### 3.6. Verification Tests

The optimal formulations obtained by the single-factor test and orthogonal test were different, so validation experiments were conducted on the two formulations. The experimental results show that the sensory scores of the optimal formula of single-factor tests and orthogonal tests were 89.5 and 88.2 points, respectively, so the optimal formula obtained by the single-factor test was selected, which was 0.4 g of NA, 10 g of white granulated sugar, an ECS to CS ratio of 20:1 (*w*/*w*), and 0.12 g of citric acid. Under this formula, the soft candy prepared had uniform texture, moderate hardness, full elasticity, chewiness, appropriate sour and sweet notes, rich red date and NA flavor, and no odor.

### 3.7. Effect of Storage Time on the Texture Characteristics of Soft Candy

**Figure 4:**
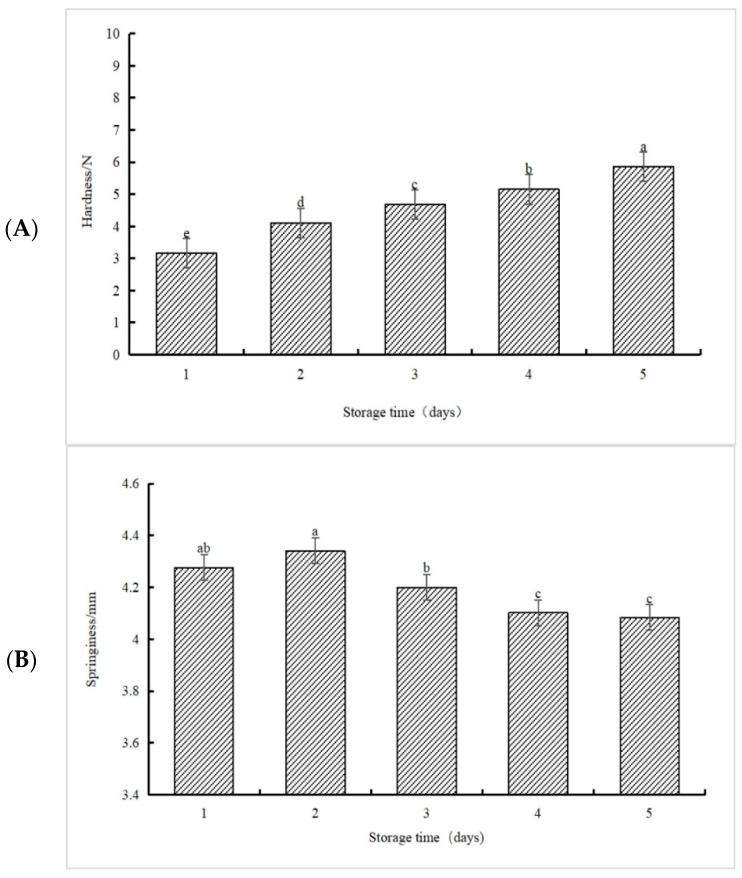

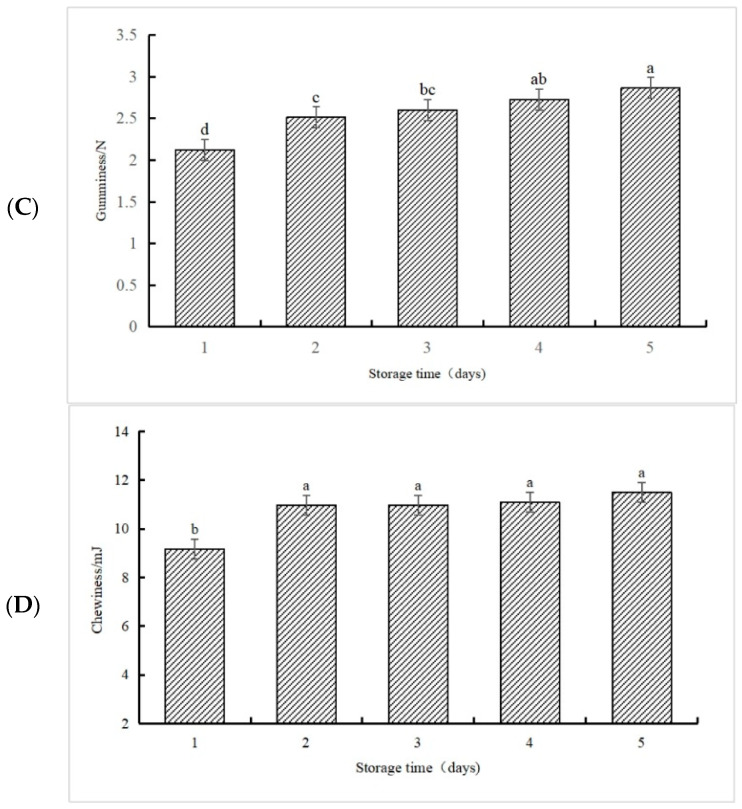
Effect of storage time on the texture characteristics of soft candy. (**A**) Effect of storage time on the hardness of soft candy. (**B**) Effect of storage time on the springiness of soft candy. (**C**) Effect of storage time on the gumminess of soft candy. (**D**) Effect of storage time on the chewiness of soft candy. Notes: different lowercase letters indicate a significant difference (*p* < 0.05).

As shown in Figure 4A–D, the hardness, gumminess, and chewiness of soft candy showed a significant upward trend overall. This was because the hardness of starchy foods in the first 24 h was mainly determined by the binding ability of amylose chains [33]. The springiness showed a significant rise, and then a decline; it had the greatest springiness on the second day of storage, which was supported by the fact that it had a good taste at that time [34]. The texture and taste of the soft candy tended to be stable when stored for two days.

## 4. Conclusions

The effects of gelatinization properties and rheological properties of CS-NA and ECS-NA were studied, and starch soft candy was developed. Rheological studies showed that both CS-NA and ECS-NA exhibited G′ > G″, evincing elastic behavior. The gelatinization results indicated that the gelatinization temperature and breakdown value of ECS-NA decreased after adding NA, while the gelatinization temperature of CS-NA increased and the breakdown value decreased. The DSC curves showed that both CS-NA and ECS-NA exhibited a single heat absorption peak. These results indicated that NA < 5% not only did not affect the rheological and gelatinizing properties of starch but also improved the viscoelasticity of starch, making it easier for the complex to form a gel; moreover, the starch was easy to gelatinize and had good stability. The soft candy obtained by 0.4 g of NA, 10 g of white granulated sugar, an ECS to CS ratio of 20:1 (21 g in total), 0.12 g of citric acid, 1 g of red jujube powder, and 16 mL of water was uniform in texture, moderate in hardness, and had good elasticity. After two days of storage, the texture of the specimens demonstrated no obvious change. These results provide a theoretical basis for the commercialization of NA.

## Figures and Tables

**Table 1:** Orthogonal test table for the optimization of NA date compound soft candies.

Level	Factor			
	A NA Addition (g)	B White Granulated Sugar Addition (g)	C Ratio of ECS to CS (g:g)	D Citric Acid Addition (g)
1	0.4	10	17:1	0.08
2	0.8	12	20:1	0.12
3	1.2	14	23:1	0.16

**Table 2:** Sensory evaluation criteria for compound soft candy.

Project	Standard for Evaluation	Sensory Score/(Score)
Color and luster (20 score)	The color was not uniform, and the transparency was low	1–11
	The color was uniform and the generally transparent	12–15
	The color was uniform, and the transparency was high	16–20
Acid sweetness (20 score)	Sweetness and sourness were not obvious or extreme	1–11
	Slightly sour or sweet	12–15
	Suitable sweetness and sourness	16–20
Form of organization (20 score)	Too soft or hard; uneven texture	1–11
	Soft or hard; the texture was basically uniform	12–15
	Moderate hardness; uniform texture	16–20
Flavor (20 score)	The taste was not delicate, and chewing was poor	1–11
	The taste was more delicate, and chewability was better	12–15
	Delicate taste and good chewing	16–20

**Table 3:** RVA gelatinization characteristic parameters of the CS-NA and ECS-NA complex systems.

Test Name	Peak Viscosity (cP)	Pasting Temperature (°C)	Setback (cP)	Breakdown (cP)	Trough Minimum Viscosity (cP)	Final Viscosity (cP)
CS	3215.47 ^f^ ± 2.64	68.73 ^b^ ± 0.11	1205.97 ^a^ ± 43.07	1698.45 ^a^ ± 4.81	1517.02 ^f^ ± 2.62	2722.99 ^d^ ± 45.27
CS+1%NA	3316.11 ^e^ ± 51.96	68.11 ^c^ ± 0.07	1096.78 ^b^ ± 71.37	1598.45 ^b^ ± 4.32	1717.66 ^e^ ± 48.52	2814.45 ^cd^ ± 31.21
CS+2%NA	3469.95 ^d^ ± 36.07	67.93 ^d^ ± 0.06	985.35 ^c^ ± 10.20	1545.38 ^c^ ± 10.4	1924.56 ^d^ ± 37.28	2909.91 ^c^ ± 36.76
CS+3%NA	3538.31 ^c^ ± 23.76	67.37 ^e^ ± 0.15	877.36 ^d^ ± 55.27	1426.86 ^d^ ± 47.65	2111.45 ^c^ ± 71.28	3041.13 ^b^ ± 119.73
CS+4%NA	3627.77 ^b^ ± 12.39	68.57 ^b^ ± 0.09	838.74 ^d^ ± 63.93	1343.37 ^e^ ± 37.3	2284.4 ^b^ ± 49.09	3123.14 ^ab^ ± 41.74
CS+5%NA	3719.77 ^a^ ± 28.88	70.06 ^a^ ± 0.08	808.24 ^d^ ± 63.21	1326.94 ^e^ ± 34.24	2392.83 ^a^ ± 28.68	3201.08 ^a^ ± 37.12
ECS	4775.12 ^a^ ± 29.43	64.96 ^a^ ± 0.32	997 ^a^ ± 61.53	2989.99 ^a^ ± 13.41	1785.14 ^a^ ± 28.28	2782.14 ^a^ ± 36.27
ECS+1%NA	4154.71 ^b^ ± 84.12	63.35 ^b^ ± 0.98	738.50 ^b^ ± 55.11	2697.79 ^b^ ± 103.35	1456.92 ^b^ ± 59.56	2195.41 ^b^ ± 114.24
ECS+2%NA	4009.75 ^b^ ± 27.63	62.8 ^b^ ± 0.77	735.10 ^b^ ± 28.51	2699.62 ^b^ ± 92.18	1310.13 ^c^ ± 96.71	2045.23 ^bc^ ± 86.94
ECS+3%NA	3622.43 ^c^ ± 40.18	62.79 ^b^ ± 1.23	628.83 ^bc^ ± 53.57	2387.45 ^c^ ± 74.44	1234.98 ^c^ ± 89.02	1863.81 ^c^ ± 140.88
ECS+4%NA	3262.40 ^d^ ± 229.66	63.52 ^b^ ± 0.13	578.12 ^c^ ± 82.16	2189.94 ^d^ ± 169.56	1072.46 ^d^ ± 61.66	1650.58 ^d^ ± 143.63
ECS+5%NA	3093.08 ^d^ ± 118.68	62.50 ^b^ ± 0.48	554.65 ^c^ ± 76.33	2123.01 ^d^ ± 105.02	970.07 ^d^ ± 25.01	1524.72 ^d^ ± 93.66

Notes: CS: corn starch; ECS: edible cassava starch; NA: *Naematelia aurantialba* powder. Each experiment was repeated three times; different superscript letters in the same column indicate a statistically significant difference (*p* < 0.05).

**Table 4:** Effects of single factors on the texture characteristics of soft candy.

Single Factor	Hardness/N	Springiness/mm	Gumminess/N	Chewiness/mJ	Sensory Score (Score)
NA addition (g)					
0	2.075 ^b^ ± 0.033	4.053 ^c^ ± 0.088	1.488 ^b^ ± 0.029	5.943 ^c^ ± 0.239	80.33 ^b^ ± 0.58
0.4	2.140 ^ab^ ± 0.699	4.224 ^ab^ ± 0.081	1.691 ^a^ ± 0.099	6.533 ^ab^ ± 0.239	83.33 ^a^ ± 1.53
0.8	2.165 ^a^ ± 0.077	4.253 ^ab^ ± 0.094	1.612 ^ab^ ± 0.131	6.602 ^b^ ± 0.461	72.00 ^c^ ± 2.00
1.2	2.203 ^a^ ± 0.025	4.276 ^a^ ± 0.153	1.595 ^ab^ ± 0.040	6.749 ^ab^ ± 0.200	69.67 ^cd^ ± 0.58
1.6	2.210 ^a^ ± 0.043	4.146 ^bc^ ± 0.067	1.538 ^b^ ± 0.740	6.782 ^ab^ ± 0.223	63.00 ^d^ ± 1.00
2.0	2.216 ^a^ ± 0.058	4.142 ^bc^ ± 0.029	1.598 ^ab^ ± 0.137	6.813 ^a^ ± 0.258	63.00 ^e^ ± 1.00
White granulated sugar addition (g)					
8	2.064 ^b^ ± 0.079	3.927 ^c^ ± 0.045	1.436 ^b^ ± 0.049	5.748 ^c^ ± 0.048	78.67 ^d^ ± 0.58
10	2.067 ^b^ ± 0.027	4.062 ^b^ ± 0.026	1.490 ^b^ ± 0.038	6.032 ^b^ ± 0.147	81.33 ^b^ ± 0.58
12	2.094 ^ab^ ± 0.091	4.064 ^b^ ± 0.040	1.467 ^b^ ± 0.072	6.073 ^b^ ± 0.197	84.33 ^a^ ± 0.58
14	2.176 ^a^ ± 0.115	4.134 ^a^ ± 0.026	1.573 ^a^ ± 0.053	6.537 ^a^ ± 0.239	82.33 ^b^ ± 0.58
16	2.096 ^ab^ ± 0.060	4.123 ^a^ ± 0.055	1.497 ^b^ ± 0.063	6.185 ^b^ ± 0.166	80.00 ^c^ ± 1.00
ECS:CS (*w*/*w*)					
14:1	2.146 ^ab^ ± 0.083	4.107 ^ab^ ± 0.048	1.468 ^a^ ± 0.057	6.033 ^a^ ± 0.269	78.67 ^d^ ± 0.58
17:1	2.101 ^ab^ ± 0.102	4.126 ^a^ ± 0.037	1.489 ^a^ ± 0.070	6.147 ^a^ ± 0.322	81.33 ^b^ ± 0.58
20:1	2.179 ^a^ ± 0.095	4.103 ^ab^ ± 0.031	1.492 ^a^ ± 0.037	6.169 ^a^ ± 0.240	82.67 ^a^ ± 0.58
23:1	2.133 ^a^ ± 0.063	4.084 ^b^ ± 0.021	1.496 ^a^ ± 0.039	6.051 ^a^ ± 0.173	81.33 ^b^ ± 0.58
26:1	2.032 ^b^ ± 0.107	4.091 ^ab^ ± 0.055	1.471 ^a^ ± 0.048	6.027 ^a^ ± 0.231	80.00 ^c^ ± 1.00
Citric acid addition (g)					
0.04	2.159 ^a^ ± 0.087	4.105 ^b^ ± 0.036	1.567 ^a^ ± 0.071	6.456 ^ab^ ± 0.233	78.33 ^c^ ± 0.58
0.08	2.229 ^a^ ± 0.094	4.166 ^a^ ± 0.008	1.585 ^a^ ± 0.055	6.666 ^a^ ± 0.212	80.67 ^b^ ± 1.15
0.12	2.218 ^a^ ± 0.126	4.132 ^ab^ ± 0.055	1.552 ^a^ ± 0.052	6.430 ^ab^ ± 0.275	82.67 ^a^ ± 0.58
0.16	2.206 ^a^ ± 0.770	4.120 ^b^ ± 0.034	1.544 ^a^ ± 0.057	6.299 ^b^ ± 0.306	80.67 ^b^ ± 0.58
0.20	2.173 ^a^ ± 0.140	4.118 ^b^ ± 0.012	1.542 ^a^ ± 0.073	6.242 ^b^ ± 0.239	78.00 ^c^ ± 1.00

Notes: CS: corn starch; ECS: edible cassava starch; NA: *Naematelia aurantialba* powder. Each experiment was repeated three times; different superscript letters in the same column indicate a statistically significant difference (*p* < 0.05).

**Table 5:** Results of the orthogonal test.

		Factors								
Test Number		A	B	C	D	Sensory Score (Score)	Hardness/N	Springiness/mm	Gumminess/N	Chewiness/mJ
1		1	1	1	1	83.67	1.93	3.89	1.35	5.25
2		1	2	2	2	86.67	1.95	4.03	1.39	5.61
3		1	3	3	3	81.33	2.03	4.16	1.46	6.08
4		2	1	2	3	78.83	2.12	4.02	1.50	6.01
5		2	2	3	1	81.83	2.69	4.23	1.80	7.10
6		2	3	1	2	79.67	2.35	4.27	1.94	7.80
7		3	1	3	2	74.00	2.31	4.02	1.61	6.48
8		3	2	1	3	78.50	2.63	4.25	1.93	8.21
9		3	3	2	1	73.67	2.58	4.29	2.23	9.58
Sensory score (score)	K_1_	83.890	78.834	80.613	79.723	A_1_B_2_C_1_D_2_				
	K_2_	80.112	82.334	79.723	80.113					
	K_3_	75.389	78.222	79.054	79.554					
	R	8.501	4.112	1.559	0.559					
Hardness/N	K_1_	1.969	2.117	2.301	2.400	A_3_B_2_C_3_D_1_				
	K_2_	2.386	2.423	2.214	2.200					
	K_3_	2.503	2.318	2.343	2.259					
	R	0.534	0.306	0.129	0.200					
Springiness/mm	K_1_	4.025	3.972	4.136	4.134	A_3_B_3_C_1_D_3_				
	K_2_	4.171	4.170	4.111	4.105					
	K_3_	4.186	4.240	4.134	4.143					
	R	0.162	0.268	0.025	0.038					
Gumminess/N	K_1_	1.400	1.487	1.739	1.793	A_3_B_3_C_1_D_1_				
	K_2_	1.746	1.706	1.707	1.649					
	K_3_	1.925	1.878	1.625	1.629					
	R	0.524	0.391	0.115	0.165					
Chewiness/mJ	K_1_	5.643	5.914	7.084	7.311	A_3_B_3_C_1_D_1_				
	K_2_	6.971	6.973	7.068	6.628					
	K_3_	8.091	7.818	6.553	6.766					
	R	2.448	1.905	0.530	0.682					

Notes: each experiment was repeated three times.

## Data Availability

Data is contained within the article.

## References

[1] Yang L., Li R., Cao Y., Li M., Luo X., Yang X., Wen S., Shen Z., Lu Q., Zi L. (2020). Research on the scientific name and taxonomic status of “Jin Er”. Edible Med. Mushrooms.

[2] Liao W., Luo Z., Liu D., Ning Z., Yang J., Ren J. (2015). Structure characterization of a novel polysaccharide from Dictyophora indusiata and its macrophage immunomodulatory activities. J. Agric. Food Chem..

[3] Sun T., Jiang H., Wang Y., Wang S., Peng W., Lei P. (2022). Research Advances on *Naematelia aurantialba* Polysaccharides. J. Chin. Inst. Food Sci. Technol..

[4] Sun T., Jiang H., Yang K., Li X., Wang S., Yao H., Wang R., Li S., Gu Y., Lei P. (2022). Nutritional Function and Flavor Evaluation of a New Soybean Beverage Based on *Naematelia aurantialba* Fermentation. Foods.

[5] Sun T., Wang R., Sun D., Li S., Xu H., Qiu Y., Lei P., Sun L., Xu X., Zhu Y. (2020). High-efficiency production of *Tremella aurantialba* polysaccharide through basidiospore fermentation. Bioresour. Technol..

[6] Hao R., Ji Z. (2021). Development of Functional *Tremella aurantialba* and Apple Compound Jam. Farm Prod. Process..

[7] He R., Sun D.-F., Luo X.-L., Zhou P., Zhang W.-S. (2022). Study on Formula Selection of Toast with *Tremella aurantialba*. Edible Fungi China.

[8] Meng L., Zhao Y., Liu J., Pan P., Liu G., Feng M., Wu Y., Yang Y. (2000). Basic Pharmacological Studies on *Tremella aurantialba* Polysaccharide-Peptide Capsule. Acta Edulis Fungi.

[9] Gao J., Zhu L., Huang J., Li L., Yang Y., Xu Y., Wang Y., Wang L. (2021). Effect of dandelion root polysaccharide on the pasting, gelatinization, rheology, structural properties and in vitro digestibility of corn starch. Food Funct..

[10] Yang F., Du Q., Miao T., Zhang X., Xu W., Jia D. (2022). Interaction between potato starch and *Tremella fuciformis* polysaccharide. Food Hydrocoll..

[11] Li J.-M., Nie S.-P. (2016). The functional and nutritional aspects of hydrocolloids in foods. Food Hydrocoll..

[12] Nawab A., Alam F., Haq M.A., Hasnain A. (2016). Effect of guar and xanthan gums on functional properties of mango (*Mangifera indica*) kernel starch. Int. J. Biol. Macromol..

[13] He J., Zeng L., Gong J., He Y., Liu X., Zhang L., Xu N., Wang Q. (2021). Effects of two contrasting dietary polysaccharides and tannic acid on the digestive and physicochemical properties of wheat starch. Food Sci. Nutr..

[14] Tian L., Hu S., Jia J., Tan W., Yang L., Zhang Q., Liu X., Duan X. (2021). Effects of short-term fermentation with lactic acid bacteria on the characterization, rheological and emulsifying properties of egg yolk. Food Chem..

[15] Li Y., Cui W., Zhao C., Wu Y., Wang S., Cao Y., Xu X., Liu J. (2021). Physicochemical and Structural Properties of Corn Starch-Auricularia cornea Ehrenb. Polysaccharide Blends. Food Sci..

[16] Liu M., Zhao X., Kan J., Zhang F., Zheng J. (2018). Effect of Xanthan Gum on Pasting, Rheological and Texture Properties of Lotus Root Starch. Food Sci..

[17] Liu C., Zhang H., Chen R., Chen J., Liu X., Luo S., Chen T. (2021). Effects of creeping fig seed polysaccharide on pasting, rheological, textural properties and in vitro digestibility of potato starch. Food Hydrocoll..

[18] Zhang Y., Gu Z., Zhu L., Hong Y. (2018). Comparative study on the interaction between native corn starch and different hydrocolloids during gelatinization. Int. J. Biol. Macromol..

[19] Luo Y., Liu X., Ke Z., Yang J., Li Y., Xie X., Li L. (2023). Insight into the improvement in pasting and gel properties of waxy corn starch by critical melting treatments. Int. J. Biol. Macromol..

[20] Wang R., Wan J., Liu C., Xia X., Ding Y. (2019). Pasting, thermal, and rheological properties of rice starch partially replaced by inulin with different degrees of polymerization. Food Hydrocoll..

[21] Zhang C., Wang Z.-J., Liu Q.-Q., Qian J.-Y., Lim S.-T. (2022). Improvement of pasting and gelling behaviors of waxy maize starch by partial gelatinization and freeze-thawing treatment with xanthan gum. Food Chem..

[22] Mahmood K., Kamilah H., Shang P.L., Sulaiman S., Ariffin F., Alias A.K. (2017). A review: Interaction of starch/non-starch hydrocolloid blending and the recent food applications. Food Biosci..

[23] Yin X., Zheng Y., Kong X., Cao S., Chen S., Liu D., Ye X., Tian J. (2021). RG- I pectin affects the physicochemical properties and digestibility of potato starch. Food Hydrocoll..

[24] BeMiller J.N. (2021). Effect of hydrocolloids on normal and waxy maize starches cross-linked with epichlorohydrin. Food Hydrocoll..

[25] Kong L., Kong M.Z., Yu J.X., Cheng D.C., Huang R., Lv Y.P. (2019). Effect of Gelatinization Treatment on Morphology, Structure and Thermal Properties of *Chenopodium quinoa* Starch. Sci. Technol. Food Ind..

[26] Zhang C., Lim S.-T. (2021). Physical modification of various starches by partial gelatinization and freeze-thawing with xanthan gum. Food Hydrocoll..

[27] Sun Z., Sun X., Ge X., Lu Y., Zhang X., Shen H., Yu X., Zeng J., Gao H., Li W. (2023). Structural, rheological, pasting, and digestive properties of wheat A-starch: Effect of outshell removal combined with annealing. Int. J. Biol. Macromol..

[28] Ma S., Zhu P., Wang M., Wang F., Wang N. (2019). Effect of konjac glucomannan with different molecular weights on physicochemical properties of corn starch. Food Hydrocoll..

[29] Ma Y.-S., Pan Y., Xie Q.-T., Li X.-M., Zhang B., Chen H.-Q. (2019). Evaluation studies on effects of pectin with different concentrations on the pasting, rheological and digestibility properties of corn starch. Food Chem..

[30] Zhang Y., Zhao X., Bao X., Xiao J., Liu H. (2021). Effects of pectin and heat-moisture treatment on structural characteristics and physicochemical properties of corn starch. Food Hydrocoll..

[31] Xu N., Zhang Y., Zhang G., Tan B. (2021). Effects of insoluble dietary fiber and ferulic acid on rheological and thermal properties of rice starch. Int. J. Biol. Macromol..

[32] Cao C.Y., Yang J.D., Liu H.C., Zou Y., Tong Z.Q., Wang D.W. (2021). Study on the gelatinization characteristics, rheological properties and extruded rice quality of Tremella-multigrain flour. J. Jilin Agric. Univ..

[33] Wang S., Li C., Copeland L., Niu Q., Wang S. (2015). Starch Retrogradation: A Comprehensive Review. Compr. Rev. Food Sci. Food Saf..

[34] Liu T., Wang K., Xue W., Wang L., Zhang C., Zhang X., Chen Z. (2021). In vitro starch digestibility, edible quality and microstructure of instant rice noodles enriched with rice bran insoluble dietary fiber. LWT.

